# (Benzophenone imine-κ*N*)­chlorido(hydrido­tripyrazolyl­borato)­(triphenyl­phosphine)ruthenium(II) diethyl ether solvate

**DOI:** 10.1107/S1600536808033722

**Published:** 2008-10-22

**Authors:** Hung-Chun Tong, Chih-Yung Chen Hsu, Yih-Hsing Lo, Chia-Her Lin, Yu Wang

**Affiliations:** a Department of Chemical Engineering, Tatung University, Taipei 104, Taiwan; b Department of Chemistry, Chung-Yuan Christian University, Chung-Li 320, Taiwan; c Department of Chemistry, National Taiwan University, Taipei 106, Taiwan

## Abstract

The reaction of RuCl(Tp)(Ph_3_P)_2_, where Tp is [(CH)_3_N_2_]_3_BH, with benzophenone imine leads to the formation of the title compound, [Ru(C_9_H_10_BN_6_)Cl(C_13_H_11_N)(C_18_H_15_P)]·C_4_H_10_O. The environment about the Ru atom corresponds to a slightly distorted octa­hedron and the bite angle of the Tp ligand produces an average N—Ru—N angle of 86.3 (9)°. The three Ru—N(Tp) bond lengths [2.117 (2), 2.079 (2) and 2.084 (2) Å] are slightly longer than the average distance (2.038 Å) in other ruthenium–Tp complexes.

## Related literature

For background literature, see: Albertin *et al.* (2008 ▶); Burrows (2001 ▶); Harman & Tube (1988 ▶); Pavlik *et al.* (2005 ▶). For related structures, see: Alock *et al.* (1992 ▶); Bohanna *et al.* (1996 ▶); Gemel *et al.* (1996 ▶); Slugovc *et al.* (1998 ▶).
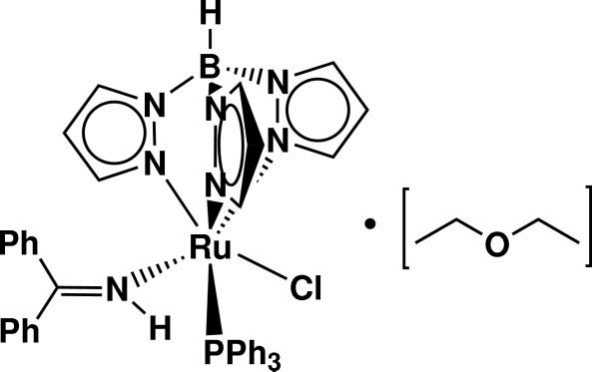


## Experimental

### 

#### Crystal data

[Ru(C_9_H_10_BN_6_)Cl(C_13_H_11_N)(C_18_H_15_P)]·C_4_H_10_O
*M* *_r_* = 867.18
Monoclinic, 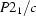

*a* = 9.3768 (1) Å
*b* = 30.1803 (5) Å
*c* = 14.9092 (2) Å
β = 96.126 (1)°
*V* = 4195.13 (10) Å^3^
*Z* = 4
Mo *K*α radiation
μ = 0.52 mm^−1^
*T* = 295 (2) K
0.20 × 0.15 × 0.10 mm

#### Data collection

Nonius KappaCCD diffractometer
Absorption correction: multi-scan (*SORTAV*; Blessing, 1995 ▶) *T*_min_ = 0.915, *T*_max_ = 0.952
28699 measured reflections
9587 independent reflections
7313 reflections with *I* > 2σ(*I*)
*R*_int_ = 0.037

#### Refinement

*R*[*F* ^2^ > 2σ(*F* ^2^)] = 0.038
*wR*(*F* ^2^) = 0.105
*S* = 1.02
9587 reflections
505 parameters
H-atom parameters constrained
Δρ_max_ = 1.60 e Å^−3^
Δρ_min_ = −0.46 e Å^−3^

### 

Data collection: *COLLECT* (Nonius, 1999 ▶); cell refinement: *DENZO* (Otwinowski & Minor, 1997 ▶) and *SCALEPACK* (Otwinowski & Minor, 1997 ▶); data reduction: *DENZO* and *SCALEPACK*; program(s) used to solve structure: *SHELXS97* (Sheldrick, 2008 ▶); program(s) used to refine structure: *SHELXL97* (Sheldrick, 2008 ▶); molecular graphics: *SHELXTL* (Sheldrick, 2008 ▶); software used to prepare material for publication: *SHELXTL*.

## Supplementary Material

Crystal structure: contains datablocks I, global. DOI: 10.1107/S1600536808033722/rk2110sup1.cif

Structure factors: contains datablocks I. DOI: 10.1107/S1600536808033722/rk2110Isup2.hkl

Additional supplementary materials:  crystallographic information; 3D view; checkCIF report

## Figures and Tables

**Fig. 1.**
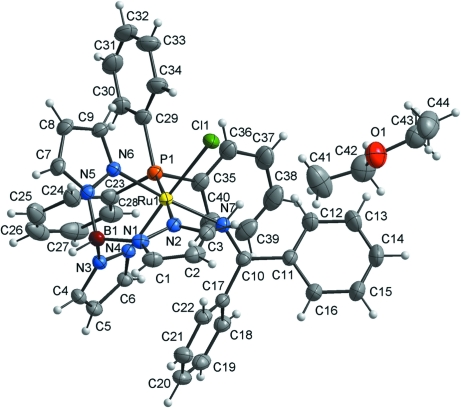
The molecular structure of (I) showing atoms numbering scheme. The displacement ellipsoids are drawn at the 30% probability level. The H atoms are drawn with arbitrary radius.

## supplementary crystallographic information

### Comment

Ruthenium(II) hydridotripyrazolylborate complexes, Ru(Tp), are of interest for stoichiometric and catalytic transformations of organic molecules (Pavlik *et al.*, 2005). The complex RuCl(Tp)(PPh_3_)_2_ (Alock *et al.*, 1992) has been used as the starting material for the synthesis of several complexes because of its substitutionally labile chloride and phosphines (Burrows, 2001). On the other hand, despite the large number of known transition metal complexes containing multidentate imine ligands, the monodentate nitrogen-bond imine derivatives are rather rare (Albertin *et al.*, 2008). This is somewhat surprising and may be partly due to the weak Lewis basicity of the imine nitrogen atom (Harman & Tube 1988). However, coordination of an imine on a metal fragment would be an important step in its activation toward nucleophilic attack or its hydrogenation reaction to give the amine.

The complex [RuCl(Tp)(PPh_3_)_2_] reacts smoothly with benzophenone imine in warm toluene affording the title compound RuCl(Tp)(PPh_3_)(HN═CPh_2_) ((I)). The complex (I) is yellow crystalline solid which in their IR spectra display one medium band near 3312 cm^-1^, attributable to ν(NH) in the imine ligand. We have observed that the benzophenone imine is lost readily in solution. It appears that a rapid dissociation equilibrium occurs which leads to the formation of variable amounts of free imine plus other species. This behavior has been observed in other imine complexes of ruthenium, as in the case of RuHCl(CO)(HN═CPh_2_)(PiPr_3_)_2_, where PiPr is tri(isopropyl)phosphine, (Bohanna *et al.*, 1996), and hence in all further operations for the purification of (I), an excess of free imine was added in order to prevent decomposition by imine ligand dissociation. In complex (I), the environment about the ruthenium metal center corresponds to a slightly distorted octahedron and the bite angle of the Tp ligand produces an average N—Ru—N angle of 86.3° only slightly distorted from 90°. The three Ru—N(Tp) bond lengths: 2.117 (2), 2.079 (2) and 2.084 (2) Å are slightly longer than the average distance of 2.038 Å in other ruthenium Tp complexes (Gemel *et al.*, 1996; Slugovc *et al.*, 1998). The Ru1—N7 and N7—C10 bond lengths of 2.063 (2) Å and 1.284 (3) Å correspond to single Ru—N and double C═N bonds. The angles (121.5 (2)°, 120.2 (3)° and 117.9 (2)°) around C10 indicate a *sp*^2^ hybridization as expected.

### Experimental

The synthesis of the title compound (I) was carried out as follows: to a solution of RuCl(Tp)(PPh_3_)_2_ (3.95 g, 4.50 mmol) in toluene (100 ml), an excess of benzophenone imine (7.9 ml, 45.0 mmol) were added. The mixture was heated using a warm water bath for 30 min. A deep yellow color developed during this time. The reaction mixture was stirred for a further 2 h at room temperature. Then, it was concentrated to approximately half of the volume and cooled to 253 K. The yellow precipitate was filtered off, washed with ethanol and ether, dried under vacuum to give the (I) (3.34 g, 95% yield). Spectroscopic analysis: IR (KBr, cm^-1^): ν(BH) 2467 cm^-1^; ν(NH) 3312 cm^-1^. The ^1^H NMR (CDCl_3_, 303 K, d, p.p.m.): δ 5.67 (t, *J*_HH_ = 2.0 Hz, 1H, Tp), 5.78 (t, 1H, *J*_HH_ = 2.0 Hz, Tp), 5.95 (d, 1H, *J*_HH_ = 2.0 Hz, Tp), 6.11 (t, 1H, *J*_HH_ = 2.0 Hz, Tp), 6.45 (d, 1H, *J*_HH_ = 2.0 Hz, Tp), 6.73–7.70 (Ph, Tp), 8.13 (d, 1H, *J*_HH_ = 2.0 Hz, Tp), 12.45 (s, 1H, HN). The ^13^C NMR (CDCl_3_, 303 K, d, p.p.m.): 105.2–148.4 (m, Ph, PPh_3_, Tp), 179.9 (s, HN═C (Ph)_2_). The ^31^P NMR (CDCl_3_, 303 K, d, p.p.m.): d 51.3. The MS (*m*/*z*, Ru 102): 793.2 (*M*^+^),758.1 (*M*^+^ - Cl), 612.2 (*M*^+^ - HN═C (Ph)_2_). Anal. Calc. for C_40_H_36_BClN_7_PRu, (%): C, 60.58; H,4.58; N, 12.36. Found (%): C, 60.43; H, 4.61; N, 12.42. The bright-yellow crystals of (I) for X-ray structure analysis were obtained by recrystallization of the crude product from dichloromethane–ether containing free benzophenone imine.

### Refinement

The H atoms were placed in idealized positions and constrained to ride on their parent atoms, with C—H = 0.93–0.98 Å and *U*_iso_(H) = 1.2 or 1.5*U*_eq_(C), N—H = 0.86 Å and *U*_iso_(H) = 1.2*U*_eq_(N), B—H = 1.10 Å and *U*_iso_(H) = 1.2*U*_eq_(B).

### Crystal data

**Table:**

[Ru(C_9_H_10_BN_6_)Cl(C_13_H_11_N)(C_18_H_15_P)]·C_4_H_10_O	*F*(000) = 1792
*M**_r_* = 867.18	*D*_x_ = 1.373 Mg m^−^^3^
Monoclinic, *P*2_1_/*c*	Mo *K*α radiation, λ = 0.71073 Å
Hall symbol: -P 2ybc	Cell parameters from 61738 reflections
*a* = 9.3768 (1) Å	θ = 1–27.5°
*b* = 30.1803 (5) Å	µ = 0.52 mm^−^^1^
*c* = 14.9092 (2) Å	*T* = 295 K
β = 96.126 (1)°	Plate, yellow
*V* = 4195.13 (10) Å^3^	0.20 × 0.15 × 0.10 mm
*Z* = 4	

### Data collection

**Table:**

Nonius KappaCCD diffractometer	9587 independent reflections
Radiation source: fine-focus sealed tube	7313 reflections with *I* > 2σ(*I*)
graphite	*R*_int_ = 0.037
φ and ω scans	θ_max_ = 27.5°, θ_min_ = 1.5°
Absorption correction: multi-scan (SORTAV; Blessing, 1995)	*h* = −12→12
*T*_min_ = 0.915, *T*_max_ = 0.952	*k* = −38→39
28699 measured reflections	*l* = −18→19

### Refinement

**Table:**

Refinement on *F*^2^	Primary atom site location: structure-invariant direct methods
Least-squares matrix: full	Secondary atom site location: difference Fourier map
*R*[*F*^2^ > 2σ(*F*^2^)] = 0.038	Hydrogen site location: inferred from neighbouring sites
*wR*(*F*^2^) = 0.105	H-atom parameters constrained
*S* = 1.02	*w* = 1/[σ^2^(*F*_o_^2^) + (0.0492*P*)^2^ + 2.3324*P*] where *P* = (*F*_o_^2^ + 2*F*_c_^2^)/3
9587 reflections	(Δ/σ)_max_ < 0.001
505 parameters	Δρ_max_ = 1.60 e Å^−^^3^
0 restraints	Δρ_min_ = −0.46 e Å^−^^3^

### Special details

**Table:**

Geometry. All s.u.'s (except the s.u. in the dihedral angle between two l.s. planes) are estimated using the full covariance matrix. The cell s.u.'s are taken into account individually in the estimation of s.u.'s in distances, angles and torsion angles; correlations between s.u.'s in cell parameters are only used when they are defined by crystal symmetry. An approximate (isotropic) treatment of cell s.u.'s is used for estimating s.u.'s involving l.s. planes.
Refinement. Refinement of *F*^2^ against ALL reflections. The weighted *R*-factor *wR* and goodness of fit *S* are based on *F*^2^, conventional *R*-factors *R* are based on *F*, with *F* set to zero for negative *F*^2^. The threshold expression of *F*^2^ > σ(*F*^2^) is used only for calculating *R*-factors(gt) *etc*. and is not relevant to the choice of reflections for refinement. *R*-factors based on *F*^2^ are statistically about twice as large as those based on *F*, and *R*-factors based on ALL data will be even larger.

### Fractional atomic coordinates and isotropic or equivalent isotropic displacement parameters (Å^2^)

**Table:**

	*x*	*y*	*z*	*U*_iso_*/*U*_eq_	
Ru1	0.29312 (2)	0.099417 (7)	0.750725 (14)	0.03648 (7)	
Cl1	0.44869 (8)	0.14260 (3)	0.66246 (5)	0.05512 (19)	
P1	0.22862 (8)	0.15893 (2)	0.83575 (5)	0.04520 (17)	
N1	0.3739 (2)	0.00419 (7)	0.72037 (16)	0.0441 (5)	
N2	0.3456 (2)	0.04306 (8)	0.67632 (14)	0.0419 (5)	
N3	0.2246 (2)	0.01635 (7)	0.84529 (15)	0.0418 (5)	
N4	0.1670 (2)	0.05607 (7)	0.81661 (14)	0.0378 (5)	
N5	0.4838 (2)	0.03736 (7)	0.86467 (15)	0.0423 (5)	
N6	0.4675 (2)	0.08098 (7)	0.84144 (15)	0.0409 (5)	
N7	0.1440 (2)	0.11552 (8)	0.64377 (15)	0.0450 (5)	
H7A	0.1717	0.1399	0.6215	0.054*	
B1	0.3757 (3)	0.00265 (10)	0.8237 (2)	0.0450 (7)	
H1	0.4047	−0.0306	0.8498	0.054*	
C1	0.3922 (3)	−0.02816 (11)	0.6606 (2)	0.0563 (8)	
H1A	0.4122	−0.0577	0.6745	0.068*	
C2	0.3764 (3)	−0.01009 (12)	0.5761 (2)	0.0620 (9)	
H2A	0.3831	−0.0246	0.5217	0.074*	
C3	0.3483 (3)	0.03437 (12)	0.5884 (2)	0.0535 (7)	
H3A	0.3334	0.0552	0.5424	0.064*	
C4	0.1277 (3)	−0.00731 (10)	0.88551 (19)	0.0498 (7)	
H4A	0.1419	−0.0354	0.9106	0.060*	
C5	0.0039 (3)	0.01730 (10)	0.8830 (2)	0.0513 (7)	
H5A	−0.0812	0.0094	0.9057	0.062*	
C6	0.0332 (3)	0.05626 (10)	0.83963 (18)	0.0439 (6)	
H6A	−0.0315	0.0794	0.8281	0.053*	
C7	0.6081 (3)	0.03204 (11)	0.9177 (2)	0.0525 (7)	
H7B	0.6429	0.0055	0.9429	0.063*	
C8	0.6744 (3)	0.07213 (11)	0.9282 (2)	0.0573 (8)	
H8A	0.7621	0.0783	0.9610	0.069*	
C9	0.5838 (3)	0.10188 (10)	0.8796 (2)	0.0506 (7)	
H9A	0.6015	0.1320	0.8744	0.061*	
C10	0.0270 (3)	0.10422 (9)	0.59664 (18)	0.0432 (6)	
C11	−0.0235 (3)	0.12837 (10)	0.51180 (19)	0.0482 (7)	
C12	0.0215 (4)	0.17094 (12)	0.4950 (2)	0.0665 (9)	
H12A	0.0822	0.1856	0.5386	0.080*	
C13	−0.0224 (5)	0.19198 (14)	0.4145 (3)	0.0849 (12)	
H13A	0.0098	0.2205	0.4039	0.102*	
C14	−0.1128 (5)	0.17104 (17)	0.3507 (3)	0.0962 (14)	
H14A	−0.1437	0.1854	0.2970	0.115*	
C15	−0.1579 (5)	0.12930 (18)	0.3653 (3)	0.1075 (18)	
H15A	−0.2178	0.1149	0.3208	0.129*	
C16	−0.1153 (4)	0.10794 (14)	0.4459 (2)	0.0787 (12)	
H16A	−0.1490	0.0796	0.4558	0.094*	
C17	−0.0629 (3)	0.06701 (10)	0.62470 (17)	0.0429 (6)	
C18	−0.2001 (3)	0.07601 (11)	0.6479 (2)	0.0524 (7)	
H18A	−0.2359	0.1047	0.6430	0.063*	
C19	−0.2831 (3)	0.04240 (13)	0.6781 (2)	0.0605 (8)	
H19A	−0.3731	0.0488	0.6957	0.073*	
C20	−0.2333 (4)	−0.00019 (13)	0.6822 (2)	0.0670 (9)	
H20A	−0.2892	−0.0227	0.7027	0.080*	
C21	−0.0998 (4)	−0.00975 (12)	0.6560 (2)	0.0638 (8)	
H21A	−0.0674	−0.0389	0.6571	0.077*	
C22	−0.0140 (3)	0.02370 (10)	0.6281 (2)	0.0515 (7)	
H22A	0.0766	0.0171	0.6117	0.062*	
C23	0.1486 (3)	0.14282 (11)	0.9387 (2)	0.0568 (8)	
C24	0.2256 (4)	0.11179 (12)	0.9944 (2)	0.0674 (9)	
H24A	0.3107	0.1003	0.9773	0.081*	
C25	0.1776 (6)	0.09801 (14)	1.0740 (3)	0.0893 (14)	
H25A	0.2316	0.0781	1.1110	0.107*	
C26	0.0504 (7)	0.11364 (18)	1.0985 (3)	0.1055 (19)	
H26A	0.0169	0.1039	1.1516	0.127*	
C27	−0.0279 (5)	0.14388 (18)	1.0444 (3)	0.0978 (16)	
H27A	−0.1145	0.1543	1.0612	0.117*	
C28	0.0211 (4)	0.15916 (13)	0.9643 (3)	0.0746 (10)	
H28A	−0.0315	0.1800	0.9288	0.090*	
C29	0.3590 (3)	0.20070 (10)	0.8864 (2)	0.0547 (7)	
C30	0.3323 (4)	0.22422 (11)	0.9633 (2)	0.0710 (9)	
H30A	0.2491	0.2187	0.9902	0.085*	
C31	0.4288 (6)	0.25585 (13)	1.0002 (3)	0.0899 (13)	
H31A	0.4091	0.2715	1.0511	0.108*	
C32	0.5529 (6)	0.26417 (14)	0.9621 (3)	0.0970 (14)	
H32A	0.6186	0.2848	0.9880	0.116*	
C33	0.5794 (4)	0.24191 (13)	0.8856 (3)	0.0882 (13)	
H33A	0.6627	0.2478	0.8590	0.106*	
C34	0.4823 (4)	0.21035 (11)	0.8474 (3)	0.0691 (9)	
H34A	0.5009	0.1957	0.7950	0.083*	
C35	0.0942 (3)	0.19466 (10)	0.7724 (2)	0.0546 (7)	
C36	0.1355 (4)	0.23402 (11)	0.7351 (2)	0.0665 (9)	
H36A	0.2295	0.2440	0.7475	0.080*	
C37	0.0374 (5)	0.25872 (13)	0.6794 (3)	0.0859 (12)	
H37A	0.0662	0.2850	0.6543	0.103*	
C38	−0.1033 (5)	0.24445 (14)	0.6609 (3)	0.0886 (13)	
H38A	−0.1688	0.2613	0.6241	0.106*	
C39	−0.1453 (4)	0.20575 (14)	0.6968 (3)	0.0815 (12)	
H39A	−0.2397	0.1962	0.6849	0.098*	
C40	−0.0466 (4)	0.18051 (12)	0.7515 (2)	0.0677 (9)	
H40A	−0.0753	0.1537	0.7743	0.081*	
O1	0.4596 (3)	0.13805 (11)	0.2598 (2)	0.0970 (9)	
C44	0.5827 (5)	0.1501 (2)	0.1320 (3)	0.128 (2)	
H44A	0.5909	0.1700	0.0826	0.192*	
H44B	0.5551	0.1213	0.1090	0.192*	
H44C	0.6734	0.1481	0.1684	0.192*	
C42	0.3529 (6)	0.15112 (18)	0.3139 (3)	0.1105 (16)	
H42A	0.2593	0.1484	0.2799	0.133*	
H42B	0.3671	0.1819	0.3313	0.133*	
C43	0.4733 (6)	0.16682 (17)	0.1874 (3)	0.1003 (14)	
H43A	0.5005	0.1961	0.2102	0.120*	
H43B	0.3819	0.1693	0.1506	0.120*	
C41	0.3591 (7)	0.12344 (18)	0.3947 (4)	0.136 (2)	
H41A	0.2859	0.1326	0.4311	0.204*	
H41B	0.4514	0.1265	0.4287	0.204*	
H41C	0.3438	0.0930	0.3775	0.204*	

### Atomic displacement parameters (Å^2^)

**Table:**

	*U*^11^	*U*^22^	*U*^33^	*U*^12^	*U*^13^	*U*^23^
Ru1	0.03305 (11)	0.03839 (12)	0.03758 (12)	−0.00223 (8)	0.00183 (8)	0.00409 (9)
Cl1	0.0477 (4)	0.0613 (4)	0.0563 (4)	−0.0130 (3)	0.0054 (3)	0.0137 (4)
P1	0.0461 (4)	0.0396 (4)	0.0488 (4)	0.0000 (3)	−0.0001 (3)	−0.0003 (3)
N1	0.0406 (11)	0.0418 (12)	0.0508 (13)	−0.0015 (9)	0.0092 (10)	−0.0054 (10)
N2	0.0369 (11)	0.0504 (13)	0.0390 (12)	−0.0029 (9)	0.0063 (9)	0.0000 (10)
N3	0.0404 (11)	0.0404 (12)	0.0448 (12)	−0.0011 (9)	0.0044 (9)	0.0060 (10)
N4	0.0360 (11)	0.0412 (12)	0.0363 (11)	−0.0017 (9)	0.0043 (9)	0.0005 (9)
N5	0.0386 (11)	0.0438 (12)	0.0439 (12)	0.0035 (9)	0.0016 (9)	0.0038 (10)
N6	0.0372 (11)	0.0418 (12)	0.0427 (12)	0.0007 (9)	0.0005 (9)	0.0019 (10)
N7	0.0414 (12)	0.0460 (12)	0.0465 (13)	−0.0009 (10)	0.0001 (10)	0.0072 (11)
B1	0.0433 (16)	0.0391 (16)	0.0526 (19)	0.0013 (13)	0.0052 (14)	0.0029 (14)
C1	0.0502 (16)	0.0511 (17)	0.069 (2)	−0.0068 (13)	0.0149 (15)	−0.0159 (16)
C2	0.0540 (18)	0.078 (2)	0.057 (2)	−0.0110 (16)	0.0188 (15)	−0.0245 (18)
C3	0.0421 (15)	0.076 (2)	0.0433 (16)	−0.0072 (14)	0.0098 (12)	−0.0031 (15)
C4	0.0509 (16)	0.0510 (16)	0.0479 (16)	−0.0085 (13)	0.0077 (13)	0.0127 (13)
C5	0.0429 (15)	0.0633 (19)	0.0490 (16)	−0.0072 (13)	0.0113 (12)	0.0066 (14)
C6	0.0356 (13)	0.0535 (16)	0.0429 (15)	−0.0007 (12)	0.0064 (11)	−0.0003 (13)
C7	0.0430 (15)	0.0604 (19)	0.0519 (17)	0.0084 (13)	−0.0049 (12)	0.0095 (14)
C8	0.0436 (15)	0.066 (2)	0.0584 (19)	−0.0009 (14)	−0.0125 (13)	0.0009 (16)
C9	0.0435 (15)	0.0518 (17)	0.0544 (17)	−0.0049 (12)	−0.0051 (13)	−0.0031 (14)
C10	0.0361 (13)	0.0507 (16)	0.0426 (15)	0.0009 (11)	0.0029 (11)	−0.0010 (12)
C11	0.0421 (14)	0.0578 (18)	0.0438 (16)	−0.0002 (13)	−0.0001 (12)	0.0047 (13)
C12	0.077 (2)	0.060 (2)	0.059 (2)	−0.0028 (17)	−0.0089 (17)	0.0092 (16)
C13	0.109 (3)	0.066 (2)	0.076 (3)	−0.006 (2)	−0.005 (2)	0.027 (2)
C14	0.101 (3)	0.112 (4)	0.069 (3)	−0.015 (3)	−0.023 (2)	0.040 (3)
C15	0.111 (3)	0.130 (4)	0.070 (3)	−0.048 (3)	−0.040 (2)	0.038 (3)
C16	0.082 (2)	0.087 (3)	0.061 (2)	−0.030 (2)	−0.0195 (19)	0.0201 (19)
C17	0.0364 (13)	0.0546 (16)	0.0371 (14)	−0.0053 (11)	0.0009 (10)	0.0007 (12)
C18	0.0389 (14)	0.0646 (19)	0.0532 (17)	0.0003 (13)	0.0030 (12)	−0.0012 (15)
C19	0.0405 (15)	0.086 (3)	0.0557 (19)	−0.0097 (16)	0.0075 (13)	−0.0034 (17)
C20	0.0551 (19)	0.079 (2)	0.067 (2)	−0.0256 (17)	0.0054 (16)	0.0059 (18)
C21	0.0590 (19)	0.0575 (19)	0.074 (2)	−0.0086 (15)	0.0038 (16)	0.0031 (17)
C22	0.0406 (14)	0.0577 (18)	0.0564 (18)	−0.0026 (13)	0.0055 (13)	−0.0014 (14)
C23	0.0644 (19)	0.0540 (18)	0.0530 (18)	−0.0114 (15)	0.0114 (15)	−0.0116 (15)
C24	0.095 (3)	0.060 (2)	0.0480 (18)	−0.0119 (18)	0.0096 (17)	−0.0069 (16)
C25	0.139 (4)	0.080 (3)	0.051 (2)	−0.026 (3)	0.020 (2)	−0.0063 (19)
C26	0.157 (5)	0.098 (4)	0.068 (3)	−0.066 (4)	0.045 (3)	−0.023 (3)
C27	0.102 (3)	0.104 (4)	0.097 (3)	−0.046 (3)	0.052 (3)	−0.054 (3)
C28	0.077 (2)	0.071 (2)	0.079 (2)	−0.0156 (19)	0.0226 (19)	−0.027 (2)
C29	0.0590 (18)	0.0418 (15)	0.0600 (19)	−0.0033 (13)	−0.0083 (14)	0.0013 (14)
C30	0.089 (2)	0.055 (2)	0.066 (2)	−0.0126 (18)	−0.0081 (18)	−0.0034 (17)
C31	0.125 (4)	0.065 (2)	0.074 (3)	−0.014 (2)	−0.017 (3)	−0.016 (2)
C32	0.109 (3)	0.065 (3)	0.108 (4)	−0.030 (2)	−0.033 (3)	−0.005 (3)
C33	0.078 (3)	0.061 (2)	0.122 (4)	−0.0238 (19)	−0.006 (2)	−0.002 (2)
C34	0.068 (2)	0.0486 (18)	0.088 (3)	−0.0090 (16)	−0.0022 (18)	−0.0045 (17)
C35	0.0554 (17)	0.0479 (17)	0.0583 (18)	0.0112 (13)	−0.0046 (14)	−0.0091 (14)
C36	0.077 (2)	0.0504 (18)	0.070 (2)	0.0082 (16)	−0.0056 (17)	0.0031 (16)
C37	0.112 (3)	0.057 (2)	0.083 (3)	0.017 (2)	−0.014 (2)	0.007 (2)
C38	0.097 (3)	0.072 (3)	0.090 (3)	0.035 (2)	−0.024 (2)	−0.007 (2)
C39	0.063 (2)	0.080 (3)	0.096 (3)	0.0233 (19)	−0.018 (2)	−0.018 (2)
C40	0.061 (2)	0.056 (2)	0.082 (2)	0.0105 (16)	−0.0078 (17)	−0.0080 (18)
O1	0.105 (2)	0.110 (2)	0.0786 (19)	0.0160 (18)	0.0204 (16)	0.0066 (18)
C44	0.099 (4)	0.197 (6)	0.090 (3)	0.006 (4)	0.019 (3)	0.020 (4)
C42	0.131 (4)	0.104 (4)	0.098 (4)	0.019 (3)	0.020 (3)	−0.021 (3)
C43	0.113 (4)	0.105 (4)	0.081 (3)	−0.007 (3)	0.002 (3)	−0.003 (3)
C41	0.210 (7)	0.095 (4)	0.115 (4)	0.027 (4)	0.075 (4)	0.007 (3)

### Geometric parameters (Å, °)

**Table:**

Ru1—N7	2.063 (2)	C18—H18A	0.9300
Ru1—N4	2.079 (2)	C19—C20	1.367 (5)
Ru1—N6	2.084 (2)	C19—H19A	0.9300
Ru1—N2	2.117 (2)	C20—C21	1.381 (5)
Ru1—P1	2.3158 (8)	C20—H20A	0.9300
Ru1—Cl1	2.4429 (7)	C21—C22	1.382 (4)
Ru1—B1	3.183 (3)	C21—H21A	0.9300
P1—C35	1.841 (3)	C22—H22A	0.9300
P1—C23	1.844 (3)	C23—C28	1.384 (5)
P1—C29	1.859 (3)	C23—C24	1.400 (5)
N1—C1	1.345 (4)	C24—C25	1.378 (5)
N1—N2	1.357 (3)	C24—H24A	0.9300
N1—B1	1.539 (4)	C25—C26	1.368 (7)
N2—C3	1.340 (3)	C25—H25A	0.9300
N3—C4	1.346 (3)	C26—C27	1.377 (7)
N3—N4	1.365 (3)	C26—H26A	0.9300
N3—B1	1.542 (4)	C27—C28	1.403 (6)
N4—C6	1.335 (3)	C27—H27A	0.9300
N5—C7	1.346 (3)	C28—H28A	0.9300
N5—N6	1.366 (3)	C29—C34	1.379 (5)
N5—B1	1.538 (4)	C29—C30	1.393 (5)
N6—C9	1.333 (3)	C30—C31	1.388 (5)
N7—C10	1.284 (3)	C30—H30A	0.9300
N7—H7A	0.8600	C31—C32	1.372 (6)
B1—H1	1.1000	C31—H31A	0.9300
C1—C2	1.367 (5)	C32—C33	1.368 (6)
C1—H1A	0.9300	C32—H32A	0.9300
C2—C3	1.384 (5)	C33—C34	1.397 (5)
C2—H2A	0.9300	C33—H33A	0.9300
C3—H3A	0.9300	C34—H34A	0.9300
C4—C5	1.375 (4)	C35—C36	1.384 (5)
C4—H4A	0.9300	C35—C40	1.390 (4)
C5—C6	1.384 (4)	C36—C37	1.390 (5)
C5—H5A	0.9300	C36—H36A	0.9300
C6—H6A	0.9300	C37—C38	1.387 (6)
C7—C8	1.362 (4)	C37—H37A	0.9300
C7—H7B	0.9300	C38—C39	1.360 (6)
C8—C9	1.386 (4)	C38—H38A	0.9300
C8—H8A	0.9300	C39—C40	1.393 (5)
C9—H9A	0.9300	C39—H39A	0.9300
C10—C17	1.491 (4)	C40—H40A	0.9300
C10—C11	1.493 (4)	O1—C43	1.403 (5)
C11—C16	1.380 (4)	O1—C42	1.406 (5)
C11—C12	1.383 (4)	C44—C43	1.472 (6)
C12—C13	1.381 (5)	C44—H44A	0.9600
C12—H12A	0.9300	C44—H44B	0.9600
C13—C14	1.360 (6)	C44—H44C	0.9600
C13—H13A	0.9300	C42—C41	1.462 (7)
C14—C15	1.354 (6)	C42—H42A	0.9700
C14—H14A	0.9300	C42—H42B	0.9700
C15—C16	1.385 (5)	C43—H43A	0.9700
C15—H15A	0.9300	C43—H43B	0.9700
C16—H16A	0.9300	C41—H41A	0.9600
C17—C22	1.384 (4)	C41—H41B	0.9600
C17—C18	1.394 (4)	C41—H41C	0.9600
C18—C19	1.383 (4)		
			
N7—Ru1—N4	98.08 (8)	C16—C15—H15A	119.8
N7—Ru1—N6	169.96 (9)	C11—C16—C15	120.6 (4)
N4—Ru1—N6	88.39 (8)	C11—C16—H16A	119.7
N7—Ru1—N2	87.74 (9)	C15—C16—H16A	119.7
N4—Ru1—N2	85.29 (8)	C22—C17—C18	119.0 (3)
N6—Ru1—N2	85.14 (8)	C22—C17—C10	121.8 (2)
N7—Ru1—P1	92.60 (7)	C18—C17—C10	119.2 (3)
N4—Ru1—P1	91.98 (6)	C19—C18—C17	120.3 (3)
N6—Ru1—P1	94.85 (6)	C19—C18—H18A	119.9
N2—Ru1—P1	177.26 (6)	C17—C18—H18A	119.9
N7—Ru1—Cl1	81.51 (6)	C20—C19—C18	120.2 (3)
N4—Ru1—Cl1	173.03 (6)	C20—C19—H19A	119.9
N6—Ru1—Cl1	91.13 (6)	C18—C19—H19A	119.9
N2—Ru1—Cl1	87.75 (6)	C19—C20—C21	120.0 (3)
P1—Ru1—Cl1	94.99 (3)	C19—C20—H20A	120.0
N7—Ru1—B1	126.89 (9)	C21—C20—H20A	120.0
N4—Ru1—B1	52.49 (8)	C20—C21—C22	120.4 (3)
N6—Ru1—B1	52.24 (8)	C20—C21—H21A	119.8
N2—Ru1—B1	51.71 (8)	C22—C21—H21A	119.8
P1—Ru1—B1	126.35 (6)	C21—C22—C17	120.0 (3)
Cl1—Ru1—B1	122.41 (6)	C21—C22—H22A	120.0
C35—P1—C23	105.29 (15)	C17—C22—H22A	120.0
C35—P1—C29	101.29 (14)	C28—C23—C24	118.7 (3)
C23—P1—C29	98.59 (14)	C28—C23—P1	125.2 (3)
C35—P1—Ru1	112.22 (10)	C24—C23—P1	116.1 (3)
C23—P1—Ru1	113.85 (10)	C25—C24—C23	121.2 (4)
C29—P1—Ru1	123.32 (11)	C25—C24—H24A	119.4
C1—N1—N2	109.8 (2)	C23—C24—H24A	119.4
C1—N1—B1	130.7 (3)	C26—C25—C24	120.0 (5)
N2—N1—B1	119.4 (2)	C26—C25—H25A	120.0
C3—N2—N1	106.3 (2)	C24—C25—H25A	120.0
C3—N2—Ru1	134.4 (2)	C25—C26—C27	119.9 (4)
N1—N2—Ru1	119.04 (16)	C25—C26—H26A	120.1
C4—N3—N4	110.0 (2)	C27—C26—H26A	120.1
C4—N3—B1	129.0 (2)	C26—C27—C28	120.9 (4)
N4—N3—B1	120.8 (2)	C26—C27—H27A	119.5
C6—N4—N3	105.9 (2)	C28—C27—H27A	119.5
C6—N4—Ru1	135.73 (19)	C23—C28—C27	119.3 (4)
N3—N4—Ru1	118.33 (15)	C23—C28—H28A	120.4
C7—N5—N6	109.3 (2)	C27—C28—H28A	120.4
C7—N5—B1	130.1 (2)	C34—C29—C30	118.1 (3)
N6—N5—B1	120.3 (2)	C34—C29—P1	121.1 (3)
C9—N6—N5	106.5 (2)	C30—C29—P1	120.8 (3)
C9—N6—Ru1	134.4 (2)	C31—C30—C29	120.7 (4)
N5—N6—Ru1	118.73 (16)	C31—C30—H30A	119.7
C10—N7—Ru1	146.0 (2)	C29—C30—H30A	119.7
C10—N7—H7A	107.0	C32—C31—C30	120.5 (4)
Ru1—N7—H7A	107.0	C32—C31—H31A	119.8
N5—B1—N1	108.3 (2)	C30—C31—H31A	119.8
N5—B1—N3	108.4 (2)	C33—C32—C31	119.5 (4)
N1—B1—N3	106.8 (2)	C33—C32—H32A	120.2
N5—B1—Ru1	68.71 (14)	C31—C32—H32A	120.2
N1—B1—Ru1	69.76 (14)	C32—C33—C34	120.4 (4)
N3—B1—Ru1	68.29 (14)	C32—C33—H33A	119.8
N5—B1—H1	110.7	C34—C33—H33A	119.8
N1—B1—H1	111.0	C29—C34—C33	120.7 (4)
N3—B1—H1	111.6	C29—C34—H34A	119.6
Ru1—B1—H1	179.2	C33—C34—H34A	119.6
N1—C1—C2	108.2 (3)	C36—C35—C40	118.2 (3)
N1—C1—H1A	125.9	C36—C35—P1	120.2 (2)
C2—C1—H1A	125.9	C40—C35—P1	121.1 (3)
C1—C2—C3	105.5 (3)	C35—C36—C37	120.3 (4)
C1—C2—H2A	127.2	C35—C36—H36A	119.9
C3—C2—H2A	127.2	C37—C36—H36A	119.9
N2—C3—C2	110.1 (3)	C38—C37—C36	120.5 (4)
N2—C3—H3A	125.0	C38—C37—H37A	119.8
C2—C3—H3A	125.0	C36—C37—H37A	119.8
N3—C4—C5	108.0 (3)	C39—C38—C37	119.8 (4)
N3—C4—H4A	126.0	C39—C38—H38A	120.1
C5—C4—H4A	126.0	C37—C38—H38A	120.1
C4—C5—C6	105.2 (2)	C38—C39—C40	119.9 (4)
C4—C5—H5A	127.4	C38—C39—H39A	120.1
C6—C5—H5A	127.4	C40—C39—H39A	120.1
N4—C6—C5	110.8 (2)	C35—C40—C39	121.3 (4)
N4—C6—H6A	124.6	C35—C40—H40A	119.4
C5—C6—H6A	124.6	C39—C40—H40A	119.4
N5—C7—C8	108.5 (3)	C43—O1—C42	113.4 (4)
N5—C7—H7B	125.7	C43—C44—H44A	109.5
C8—C7—H7B	125.7	C43—C44—H44B	109.5
C7—C8—C9	105.6 (3)	H44A—C44—H44B	109.5
C7—C8—H8A	127.2	C43—C44—H44C	109.5
C9—C8—H8A	127.2	H44A—C44—H44C	109.5
N6—C9—C8	110.1 (3)	H44B—C44—H44C	109.5
N6—C9—H9A	124.9	O1—C42—C41	110.2 (4)
C8—C9—H9A	124.9	O1—C42—H42A	109.6
N7—C10—C17	121.5 (2)	C41—C42—H42A	109.6
N7—C10—C11	120.6 (2)	O1—C42—H42B	109.6
C17—C10—C11	117.9 (2)	C41—C42—H42B	109.6
C16—C11—C12	117.7 (3)	H42A—C42—H42B	108.1
C16—C11—C10	120.1 (3)	O1—C43—C44	110.3 (4)
C12—C11—C10	122.1 (3)	O1—C43—H43A	109.6
C13—C12—C11	121.1 (3)	C44—C43—H43A	109.6
C13—C12—H12A	119.5	O1—C43—H43B	109.6
C11—C12—H12A	119.5	C44—C43—H43B	109.6
C14—C13—C12	120.0 (4)	H43A—C43—H43B	108.1
C14—C13—H13A	120.0	C42—C41—H41A	109.5
C12—C13—H13A	120.0	C42—C41—H41B	109.5
C15—C14—C13	120.2 (4)	H41A—C41—H41B	109.5
C15—C14—H14A	119.9	C42—C41—H41C	109.5
C13—C14—H14A	119.9	H41A—C41—H41C	109.5
C14—C15—C16	120.4 (4)	H41B—C41—H41C	109.5
C14—C15—H15A	119.8		
